# Serine integrase chimeras with activity in *E. coli* and HeLa cells

**DOI:** 10.1242/bio.20148748

**Published:** 2014-09-12

**Authors:** Alfonso P. Farruggio, Michele P. Calos

**Affiliations:** Department of Genetics, Stanford University School of Medicine, 300 Pasteur Drive, Stanford, CA 94305-5120, USA

**Keywords:** Phage integrase, Sequence-specific recombination, Chimeric recombinase, Hybrid protein, Genome engineering

## Abstract

In recent years, application of serine integrases for genomic engineering has increased in popularity. The factor-independence and unidirectionality of these large serine recombinases makes them well suited for reactions such as site-directed vector integration and cassette exchange in a wide variety of organisms. In order to generate information that might be useful for altering the specificity of serine integrases and to improve their efficiency, we tested a hybridization strategy that has been successful with several small serine recombinases. We created chimeras derived from three characterized members of the serine integrase family, phiC31, phiBT1, and TG1 integrases, by joining their amino- and carboxy-terminal portions. We found that several phiBT1-phiC31 (BC) and phiC31-TG1 (CT) hybrid integrases are active in *E. coli*. BC chimeras function on native *att*-sites and on *att*-sites that are hybrids between those of the two donor enzymes, while CT chimeras only act on the latter *att*-sites. A BC hybrid, BC{−1}, was also active in human HeLa cells. Our work is the first to demonstrate chimeric serine integrase activity. This analysis sheds light on integrase structure and function, and establishes a potentially tractable means to probe the specificity of the thousands of putative large serine recombinases that have been revealed by bioinformatics studies.

## INTRODUCTION

**Fig. 1.**
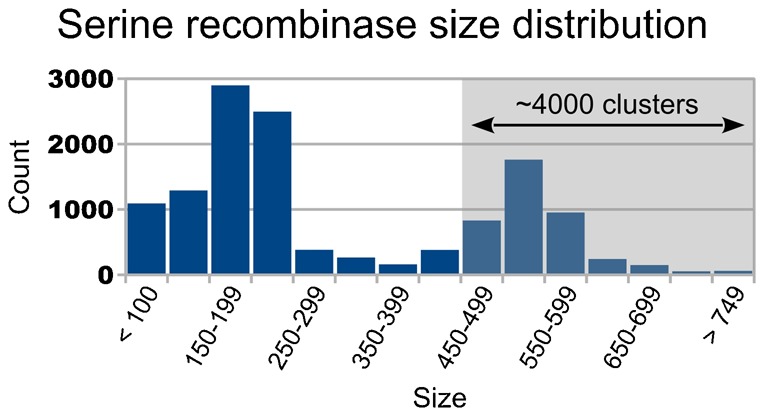
Histogram of serine recombinase lengths. All proteins with an InterPro (Hunter et al., 2012) serine recombinase catalytic domain (IPR006119; 35,076 entries) were clustered (13,019 clusters). The mean protein length of each cluster was computed, and the distribution of these lengths is presented here as a histogram. The list of putative serine recombinases was assembled with a custom script that scanned the entire InterPro “Protein matched complete” XML flatfile (∼75 GiB uncompressed; downloaded on Feb. 14, 2014) for proteins with an IPR006119 domain (35,076 proteins found). Protein sequences were downloaded from UniProt (UniProt Consortium, 2012) and were validated via CRC64 checksum comparison with InterPro. CD-HIT (Li and Godzik, 2006) version 4.6.1 was used to perform the clustering with the following parameters: 95% identity cutoff, 95% size cutoff, five character word size. Because the smallest characterized serine integrases (A118 and U153, accession numbers Q9T193 and Q8LTD8, respectively) are both 452 residues in length, we estimate that there are at least 4,000 unique putative large serine recombinases in the InterPro database as of February 14, 2014.

Serine integrases mediate recombination between two distinct ∼50 bp phage and bacterial sequences named *att*P and *att*B, respectively (Brown et al., 2011; Smith et al., 2010). Without assistance from other proteins, the reaction proceeds in a unidirectional manner to produce the left and right attachment sites – *att*L and *att*R (Smith et al., 2010). Because they are ∼200–350 residues larger than the small serine recombinases (Fig. 1), serine integrases are classified as members of the large serine recombinase sub-family (Smith and Thorpe, 2002). All serine integrases characterized to date appear to consist of an ∼120 amino acid N-terminal domain that is connected via a ∼30 residue alpha-helix to a ∼300–450 amino acid C-terminal domain (supplementary material Table S1). The N-terminal domain is principally involved in catalysis, but also imparts some sequence specificity, and the C-terminal domain appears to be primarily responsible for DNA-binding and directionality control (Ghosh et al., 2005; Gordley et al., 2007; Mandali et al., 2013; McEwan et al., 2009; Rowley et al., 2008). At present, there are thousands of putative large serine recombinases in the sequence databases (Fig. 1).

Despite their prokaryotic origins, a few serine integrases have been shown to function in a variety of plant and animal species (Chompoosri et al., 2009; Groth et al., 2000; Huang et al., 2011; Keravala et al., 2006a; Nkrumah et al., 2006; Olivares et al., 2001; Smith et al., 2010; Thomson et al., 2012; Yamaguchi et al., 2011). The most commonly used member of this group – phiC31 integrase – has been used in a multitude of studies to carry out site-directed plasmid integration and/or cassette exchange in eukaryotes (Smith et al., 2010). To date, its most popular application has been in Drosophila, where high efficiency (up to 60%) site-specific integration is possible at certain loci, and large-vector (>100 kb) integration has been accomplished (Bischof et al., 2007; Venken et al., 2006). phiC31 integrase has also been used for integration into endogenous “pseudo sites” in certain cellular environments, however it does not appear to be universally capable of this reaction (Chalberg et al., 2006; Groth et al., 2004; Ma et al., 2006; Ni et al., 2012; Olivares et al., 2002). Another serine integrase that has been applied successfully in several eukaryotic environments is Bxb1 integrase, which is notable for its high efficiency and specificity (Campbell et al., 2010; Keravala et al., 2006a; Nkrumah et al., 2006; Russell et al., 2006; Thomson et al., 2012; Yamaguchi et al., 2011; Zhao et al., 2014; Zhu et al., 2014).

To use serine integrases in eukaryotes for site-directed integration or cassette exchange – the two most popular applications – it is currently necessary to pre-introduce at least one of the wild-type sites into the host genome. This is a consequence of the fact that none of the ∼50 bp *att*-sites for the tens of characterized serine integrases are likely to exist in the host genome of interest (Brown et al., 2011).

Insertion of a wild-type *att*-site into the host genome of interest is usually accomplished via transposase-mediated insertion or random integration (Bateman et al., 2006; Belteki et al., 2003; Monetti et al., 2011). Homologous recombination has also been used to place *att*-sites (Tasic et al., 2011; Wei et al., 2011; Zhu et al., 2014), and this method is likely to see increased usage with the advent of transcription activator-like effector (TALE) and CRISPR/Cas nucleases (Christian et al., 2010; Cong et al., 2013; Mali et al., 2013). Most *att*-site introduction steps are followed by a marker screen (e.g. GFP) or selection (e.g. G418-resistance) to isolate candidate clones. To identify desirable lines, additional screens are usually performed after outgrowth, e.g. for *att*-site platform integrity, copy number, integration locus, etc. So, in addition to precluding the application of serine integrases for *in vivo* applications, *att*-site pre-introduction often consumes a considerable amount of labor and time.

If it were possible to develop integrases that specifically and efficiently recognize desired endogenous sequences, the aforementioned *att*-site introduction work could be avoided. This development would reduce the time and resources needed for tasks like site-directed integration and would make it feasible to use serine integrases for tasks like *in vivo* gene therapy. Attempts have been made to realize this goal via directed evolution of phiC31 integrase, but were ultimately not able to achieve significant alteration of specificity (Keravala et al., 2009; Sclimenti et al., 2001).

Hybridization is an alternative to directed evolution that has successfully changed the specificity of several small serine recombinases. These enzymes consist of an N-terminal catalytic domain that is similar to the corresponding serine integrase domain, a long alpha-helix linker and a C-terminal helix–turn–helix DNA binding motif (Smith and Thorpe, 2002; Yuan et al., 2008). The simplicity and modularity of these recombinases has not only made it possible to change their specificity via C-terminal domain swaps (Avila et al., 1990; Schneider et al., 2000), but also through fusion with heterologous zinc-finger and TALE DNA binding domains (Akopian et al., 2003; Mercer et al., 2012; Nomura et al., 2012).

Hybridization has yet to be attempted with serine integrases, so we decided to pursue this approach to explore enzyme function and modularity and as a method to alter specificity. We focused on the phiC31 family of enzymes – phiC31, phiBT1 and TG1 integrases – because they are currently the best characterized set of closely related serine integrases (Brown et al., 2011; Gregory et al., 2003; Morita et al., 2009; Thorpe and Smith, 1998). We constructed several binary hybrids using arrangements that involve some portion of phiC31 integrase, and looked for activity in *E. coli* and/or HeLa cells with inversion reporter assays. Specifically, we built phiC31-phiBT1 (CB), phiBT1-phiC31 (BC), phiC31-TG1 (CT) and TG1-phiC31 (TC) chimeras.

We report here that hybrids from three of the four tested architectures – BC, CT and TC chimeras – are active in *E. coli* on hybrid and/or parental *att*-sites. We also show that BC hybrids can function efficiently in HeLa cells, in both extra-chromosomal and pseudo site assays. Our study is the first to describe active serine integrase chimeras and sheds light on the structure–function relationships of these enzymes. The work also lays the groundwork for a more tractable means to sample the specificity of the putative large serine recombinases that have been identified by genome sequencing, which currently number in the thousands (Fig. 1).

## RESULTS

### Hybrid integrase naming scheme

**Fig. 2.**
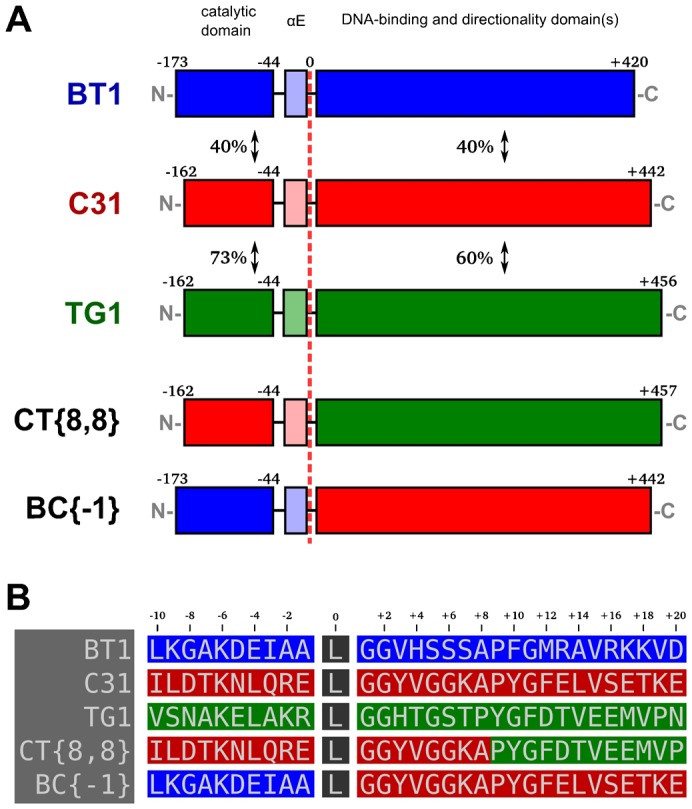
Hybrid integrases: architectures and naming. (A) Domain organization of parental and chimeric integrases. Pairwise phiBT1-phiC31 and TG1-phiC31 integrase domain alignments were performed, and the percent similarity is shown between the respective regions. EMBOSS Needle with the BLOSUM62 scoring matrix was used for all sequence alignments (Rice et al., 2000). The architectures of two representative hybrids are displayed below the parental enzymes. All chimeric integrases described in this study have been assigned systematic names that specify the parental proteins and fusion indices used to create them. (B) Detailed view of the relative indexing scheme that we developed to specify protein fusions. See the text for an explanation of our naming system.

The chimeric integrases described in this study have been assigned systematic names that specify the parental proteins and fusion indices used to create them (Fig. 2A). Our naming scheme follows the format “XY-{i,j}”, where *X* and *Y* indicate the source of the N- and C-terminal integrase sequences, and *i* and *j* specify residue positions in the N- and C-terminal parental enzymes that were connected to make the hybrid. The letters “B”, “C” and “T” are used to specify the phiBT1, phiC31 and TG1 integrases, respectively. To permit concise numbering, we use a relative index for *i* and *j* that is centered at the predicted end of alpha-helix E (αE) (Yuan et al., 2008) for each integrase (Fig. 2B). For further brevity, *j* is omitted when it is equal to *i*+1. Thus, we call the phiC31-TG1 integrase chimera where the eighth amino acids are linked “CT{8,8}”, and the phiBT1-phiC31 hybrid where phiBT1 residue −1 is connected to the zeroth phiC31 residue is named “BC{−1}” (instead of “BC{−1,0}”). Jpred 3 (Cole et al., 2008) was used for secondary structure prediction and it predicted that the following residues would terminate αE in phiBT1, phiC31 and TG1 integrase, respectively: Leu-174, Leu-163 and Leu-163.

### System for naming hybrid att-sites

**Fig. 3.**
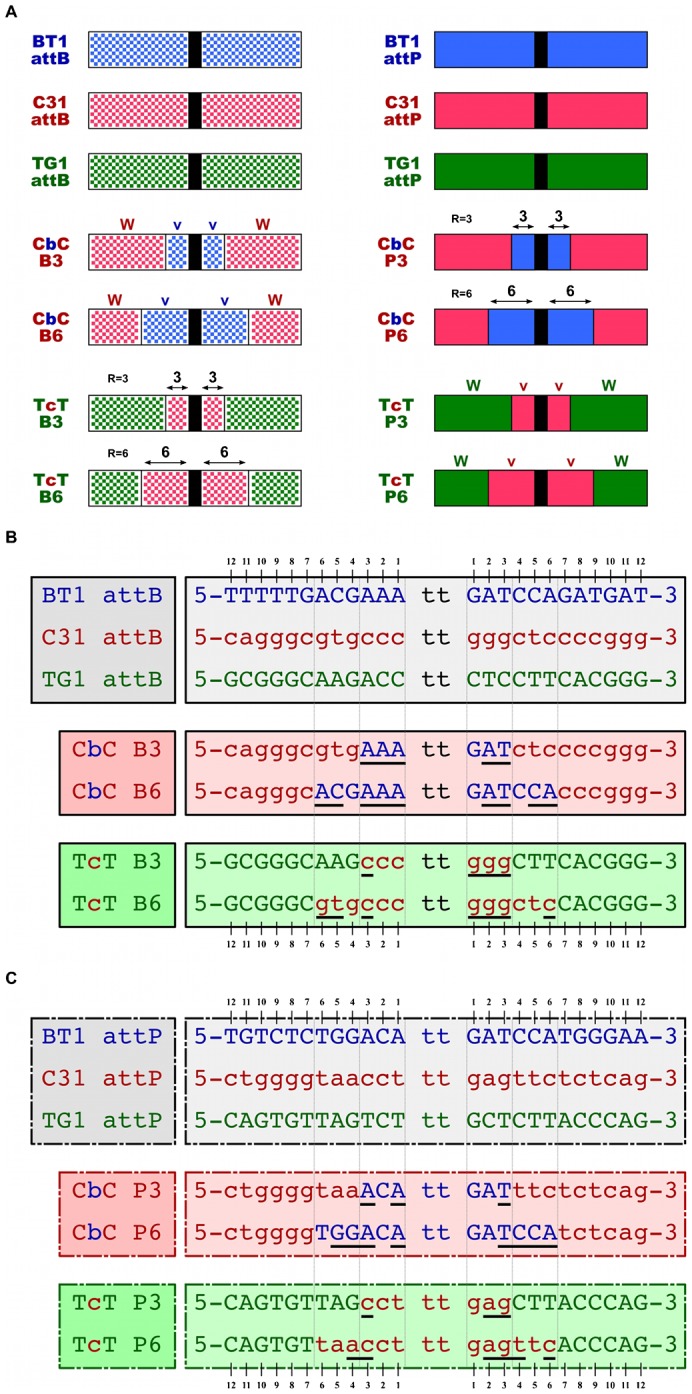
Chimeric *att*-sites: organization and nomenclature. (A) Overview of parental and hybrid *att*-site structure. Serine integrase *att*-sites consist of a dinucleotide core (black bar) that is flanked by two half-sites. Checkered patterns are used here to indicate *att*B half-sites; solid colors are used for *att*P. Drawings are not to scale. See the text for an explanation of our hybrid *att*-site naming system. (B) Detailed overview of parental and hybrid *att*B seqences. Mismatches in the B0–B3 and B0–B6 alignments are underlined. CbC B0 and TcT B0 are equivalent to the phiC31 and TG1 *att*B sites, respectively. (C) Detailed overview of parental and hybrid *att*P seqences. Mismatches in the P0–P3 and P0–P6 alignments are underlined. CbC P0 and TcT P0 are equivalent to the phiC31 and TG1 *att*P sites, respectively. In (B) and (C), only a central 26 nucleotide window is shown; see supplementary material Table S2 for the full sequences of all *att*-sites used in our study.

Chimeric *att*-sites were named using the format “WvW QR”. In this scheme, *W* and *v* refer to the source of the outer and inner *att*-site sequence, respectively (Fig. 3A). Parental sources are indicated using the same ‘B’, ‘C’ and ‘T’ code established for integrase hybrids (phiBT1, phiC31 and TG1, respectively). *Q* indicates the type of *att*-site; the letters ‘P’ and ‘B’ are used to specify *att*P and *att*B, respectively. *R* is the number of “core” bases in each *W* half-site that have been derived from the integrase *v* site (Fig. 3A–C). The dinucleotide crossover bases are not included in the tally for *R*. Thus, “CbC B3” is a phiC31-phiBT1-phiC31 hybrid *attB* sequence where the three core bases in each half-site have been taken from phiBT1 *attB*, and all remaining bases are from phiC31 attB (supplementary material Table S2; Fig. 3B). All “P0” and “B0” sites have wild-type half-sites and a ‘TT’ dinucleotide crossover core. All hybrid att-site sequences have been provided in supplementary material Table S2.

### CT hybrids are active in E. coli

**Fig. 4.**
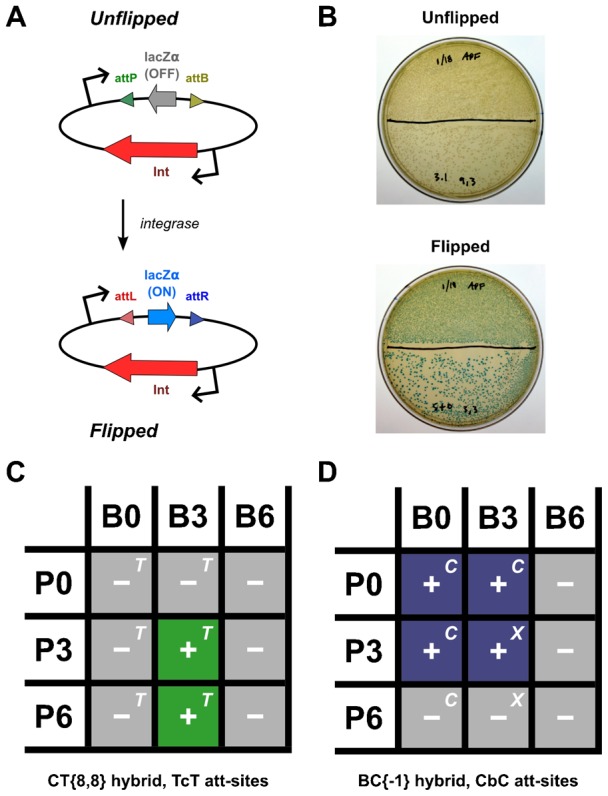
*E. coli* activity assay and results. (A) Simplified diagram of detection scheme for integrase activity. In the un-flipped state, the lacZα fragment is not expressed, as its ORF is inverted relative to the upstream promoter. Expression of lacZα occurs when the flanking *att*-sites are recombined. (B) To detect active integrases, we grew transformed *E. coli* on plates with X-gal to detect alpha-complementation of beta-galactosidase. (C) Summary of results for recombination attempts with the CT{8,8} hybrid and TcT *att*-sites. See the text for an explanation of hybrid *att*-site nomenclature. Active and inactive *att*-site pairings are indicated with ‘+’ and ‘−’, respectively. TG1 integrase is able to recombine pairings with an inset “T”. No recombination of the TcT B6 hybrid site was attempted with TG1 integrase. (D) Results for recombination of CbC *att*-site pairings with BC{−1} hybrid. Wild-type phiC31 int is able to recombine pairings with an inset “C”, but not those marked with “X”. No recombination of the CbC B6 hybrid site was attempted with phiC31 integrase. Results from one of three independent representative trials was used to construct each result summary.

In an effort to obtain functional phiC31-TG1 (CT) hybrid integrases, we constructed many fusions between the two enzymes. In total, eleven CT chimeras were constructed (supplementary material Table S3). The majority of these CT hybrids formed aggregates and/or were not active on the tested parental or chimeric *att*-sites (supplementary material Table S3). However, three chimeras, CT{−75}, CT{−41} and CT{8,8} (Fig. 2; supplementary material Table S3), showed clear signs of activity in our *E. coli* assay (Fig. 4A,B; supplementary material Fig. S1A). These hybrids seemed to function only on chimeric TcT *att*-sites (Fig. 3, Fig. 4C) that were derived from the parental *att*P and *att*B sequences (supplementary material Table S2). The CT{8,8} hybrid had the broadest activity of the three and was specifically able to recombine TcT P3 × B3 and TcT P6 × B3 (Fig. 4C). None of the functional CT hybrids were able to recombine the parental B0 or TcT B6 sites with any of the tested *att*P pairings (P0, P3, P6; Fig. 4C). TG1 integrase recombined all tested TcT B0 and B3 pairings; TcT B6 recombination was not attempted with TG1 (Fig. 4C).

### Limited TC hybrids function in E. coli

Despite our successes with CT hybrids and the strong sequence similarity of phiC31 and TG1 integrase (Fig. 2A), we did not observe any activity from TC chimeras with a complete TG1 integrase catalytic domain (residues −162 through −44; Fig. 2A; supplementary material Table S3). TC hybrids with up to ∼70% of the TG1 domain – TC{−135}, TC{−112} and TC{−80} – were active on wild-type phiC31 *att*-sites in our *E. coli* assay (supplementary material Table S3; CtC *att*-sites not tested). However, chimeras with larger swaps, like TC{−43}, TC{−7}, etc., were unable to recombine wild-type phiC31 or hybrid CtC *att*-sites (supplementary material Table S2).

### BC hybrids are active in E. coli

Because the phiBT1 and phiC31 integrases have fewer conserved regions, only three BC hybrids were constructed, all with fusions near the predicted end of α-helix E (supplementary material Table S3). Fusions were made in this region to mirror the precedent set by zinc-finger small-serine recombinase chimeras (Akopian et al., 2003). We found that two of the three chimeras – BC{−9,−6} and BC{−1} – were active in *E. coli*. Additionally, we observed that a BC hybrid, BC{−1}, was able to recombine a broader range of *att*-sites than the CT{8,8} chimera (Fig. 4D). Specifically, in addition to recombination of the CbC P3 and B3 sites, BC{−1} was active on the parental (P0 × B0) and certain hybrid-parental *att*-site combinations (P0 × B3, P3 × B0; Fig. 4D). However, this chimera did not appear to be able to recombine the P6 or B6 CbC hybrid sites.

Unlike TG1 integrase, the hybrid *att*-site activity of phiC31 integrase did not completely eclipse its hybrid offshoot. Like BC{−1}, phiC31 integrase can recombine the CbC P0 × B0, P0 × B3 and P3 × B0 pairings, however it faltered on the hybrid-hybrid CbC P3 × B3 reaction (Fig. 4D). Another deviation between the two enzymes was on the CbC P6 × B0 pairing, where phiC31 integrase was able to complete the recombination, but BC{−1} was not (Fig. 4D). Reactions involving the CbC B6 site were not attempted with phiC31 integrase.

### Integrase activity assay in HeLa

**Fig. 5.**
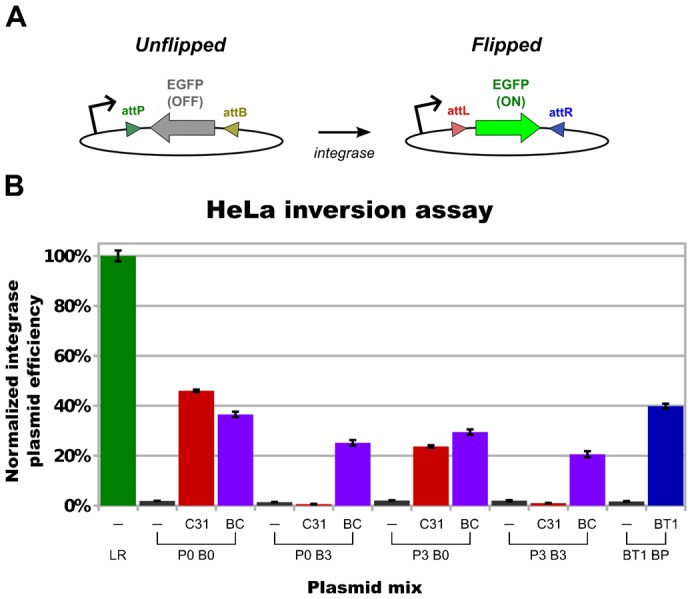
HeLa activity assay and results. (A) EGFP inversion test for integrase expression vector activity. In the starting substrate plasmid, EGFP is not significantly expressed because its ORF is inverted relative to the upstream promoter. If the flanking *att*-sites are recombined by an active integrase, EGFP expression is triggered. (B) Normalized integrase expression vector inversion efficiency. HeLa cells were transfected with different combinations of a protein expression plasmid and an EGFP inversion vector. The abbreviations “−”, “C31”, “BC”, and “BT1” refer to the negative-control, phiC31, BC{−1} and phiBT1 integrase expression plasmids, respectively. “P0”, “P3”, “B0” and “B3” all refer to the respective CbC *att*-sites. To calculate the normalized efficiency for each plasmid combination, we divided the percentage of EGFP-positive cells by the mean transfection efficiency of the positive control inversion plasmid (“LR”, pMF-CLCR; supplementary material Fig. S1E). In all trials, each plasmid combination was transfected in triplicate. The error bars indicate the standard error of the mean. Data from one of three independent representative trials is shown here.

To detect recombination in HeLa, we cloned several CMV-driven EGFP-inversion plasmids (Fig. 5A; supplementary material Fig. S1B) with various combinations of hybrid and wildtype *att*-sites. To express the chimeric and parental integrases, we utilized a tetracycline inducible (tet) promoter (supplementary material Fig. S1C,D).

To facilitate comparison of integrase expression plasmid performance, results from the inversion activity assays performed in HeLa have been normalized to the transfection efficiency of the positive-control plasmid pMF-CLCR (supplementary material Fig. S1E). Representative flow-cytometry plots of cells that have been exposed to control and experimental plasmid combinations have been provided in the supplement (supplementary material Fig. S2).

The normalization that we have applied is only valid if the following assumptions are correct: (i) the unflipped pMF plasmids transfect with similar efficiencies as the flipped pMF-CLCR vector and (ii) EGFP transcripts on flipped pMF plasmids are transcribed and translated with similar efficiencies as the pMF-CLCR EGFP transcript. We believe that both of these assumptions are likely to be true because the tested pMF plasmids share at least ∼98% sequence identity with pMF-CLCR (supplementary material Table S4; Fig. S1E). Nevertheless, even if either of these normalization assumptions proves to not be the case, our measurements in HeLa will still serve as the first qualitative confirmation of hybrid serine integrase activity in a mammalian cell line.

While the pMF plasmids consist of nearly identical sequence segments, the integrase expression vector sequences differ by ∼5–25% (supplementary material Table S4). This variation stems completely from sequence differences between the integrase ORFs, as the promoter, untranslated regions and plasmid backbone employed in these plasmids are all identical. Thus, despite the fact that we have transfected equimolar amounts of these vectors, the integrase sequence differences may result in different transcription efficiencies, transcript stabilities and/or translation efficiencies. Therefore, it is our intention that the efficiencies that we report here be interpreted as integrase plasmid efficiencies, and not intrinsic enzyme efficiencies.

### BC{−1} hybrid functions in HeLa

To assess hybrid serine integrase activity in a mammalian environment, we tested a BC hybrid, BC{−1}, in HeLa cells. As was the case in *E. coli*, BC{−1} integrase demonstrated clear activity on hybrid and wild-type *att*-sites in HeLa (Fig. 5B). The BC{−1} expression vector performed best on wild-type phiC31 *att*-sites (P0 × B0, 37%), and led to recombination of all tested hybrid *att*-site pairings at the ∼20% efficiency level or above: P0 × B3 (25%), P3 × B0 (29%), P3 × B3 (21%).

In HeLa, phiC31 integrase was capable of performing the P3 × B0 hybrid recombination and its native P0 × B0 reaction (24% and 46% eff., respectively; Fig. 5B). Despite its ability to mediate P0 × B3 recombination in *E. coli* (Fig. 4D), we did not observe significant phiC31 integrase activity for this pairing in HeLa. In both environments, phiC31 integrase was unable to perform P3 × B3 recombination.

### BC{−1} hybrid recognizes pseudo sites in HeLa

**Fig. 6.**
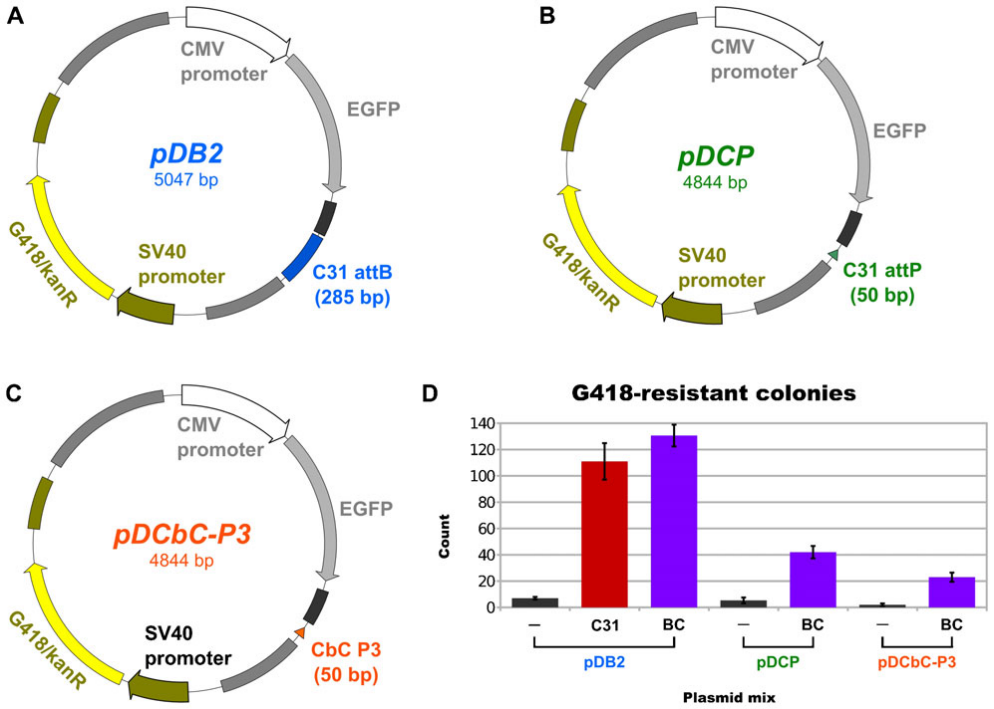
HeLa pseudo site integration assay. (A–C) Maps of pDB2, pDCP and pDCbC-P3 donor plasmids. (D) Mean counts of G418-resistant colonies. HeLa cells were transfected with different combinations of an integrase expression vector and a donor plasmid, and were then subjected to G418 selection. The abbreviations “−”, “C31” and “BC” refer to the negative control, phiC31 and BC{−1} integrase expression plasmids, respectively. The pDB2, pDCP and pDCbC-P3 vectors carry a 285 bp phiC31 *att*B, 50 bp phiC31 *att*P and 50 bp CbC P3 site, respectively. In all trials, each plasmid combination was transfected in triplicate. The error bars indicate standard error of the mean. Data from one of two independent representative trials is shown here.

Because the phiC31 and phiBT1 integrases are capable of pseudo *att*-site integration in mammalian cells (Chalberg et al., 2006) (phiBT1 int: unpublished observations), we next tested whether BC{−1} integrase could perform this function in HeLa cells. To detect pseudo site integration, we co-transfected different combinations of G418-resistance donor plasmids, which carry an *att*-site (Fig. 6A–C), with integrase expression vectors, and then counted the resistant colonies present after two weeks of G418 selection. A low donor:integrase plasmid ratio was used to minimize the contribution of random integrants to our colony counts.

In line with its parental enzymes, we found that BC{−1} integrase was able to carry out pseudo site integration in HeLa (Fig. 6D). Relative to phiC31 integrase, BC{−1} efficiently integrated a phiC31 *att*B donor (pDB2) into pseudo *att*P sites (Fig. 6D). BC{−1} also proved to be capable of pseudo *att*B recombination, as it mediated integration of CbC P0 and P3 donor plasmids (pDCP and pDCbC-P3, respectively) into the genome (Fig. 6D). The CbC B3 vector could not be integrated by either enzyme, and phiC31 integrase was not able to recombine the CbC P0 or P3 donors into pseudo *att*B sites.

## DISCUSSION

**Fig. 7.**
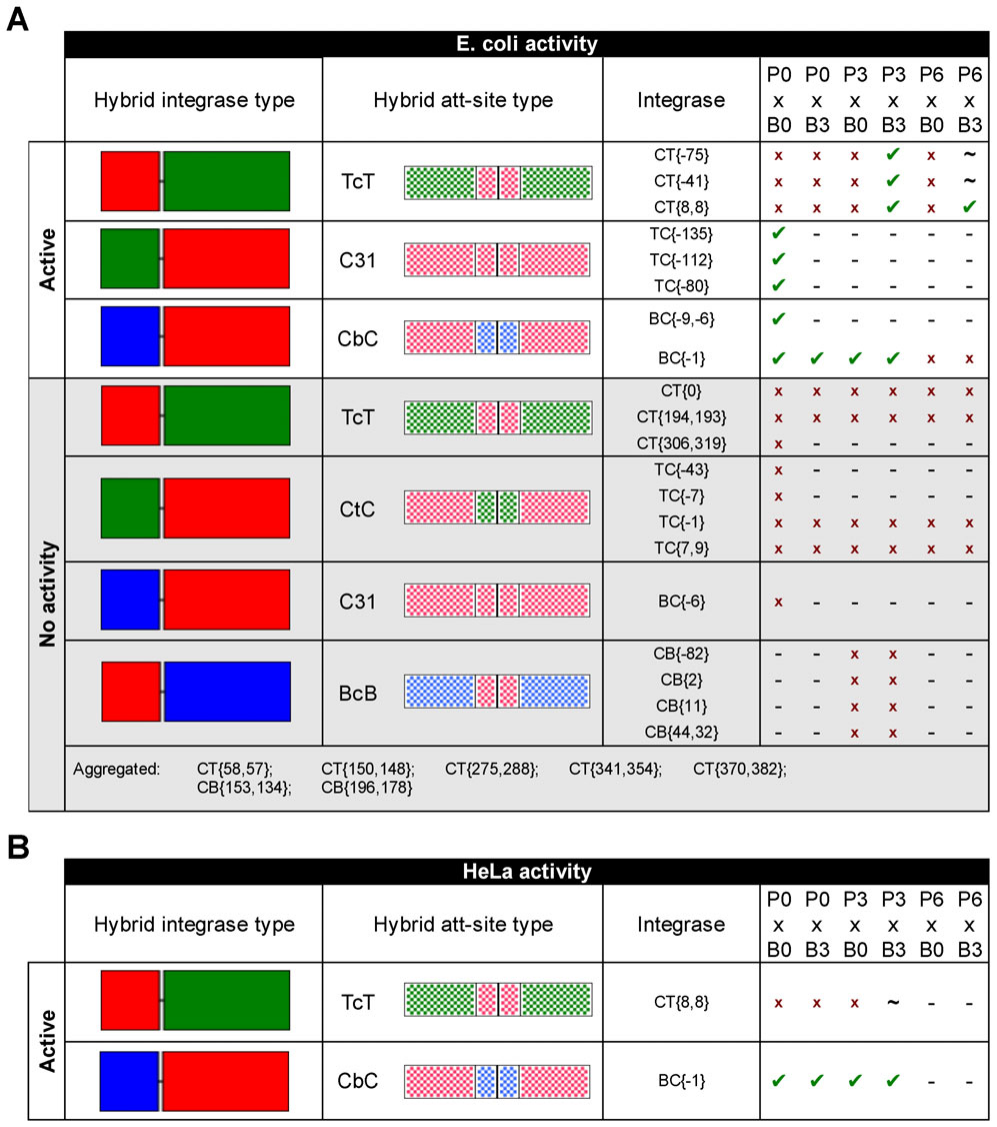
Summary of hybrid integrase activity results. (A) Hybrid activity in E. coli. Chimeric integrases have been grouped according to their activity and type. Active hybrids were able to perform recombination on at least one of the indicated att-site pairings, while inactive hybrids were not. Positive recombination results are indicated with a checkmark and negative results are marked with an “x”. Untested combinations are denoted with a dash (“−”) and pairings that produced weak positive results (light blue colonies) are marked with a tilde (“∼”). Hybrids that aggregated when overexpressed in E. coli were not subjected to any recombination assays. (B) Hybrid activity in HeLa. The CT{8,8} integrase TcT P3 × B3 reaction is marked with a tilde because the recombination product could only be detected via PCR.

In this study, we have demonstrated that it is possible to construct functional serine integrase hybrids. We show that they can operate in *E. coli* on parental and/or chimeric *att*-sites, and that a select few are also able to function in HeLa cells. Overall, three of the four attempted hybrid architectures yielded chimeras with at least marginal activity in *E. coli* (CT, TC and BC; Fig. 7A). However, only two of these hybrid enzyme classes supported full catalytic domain substitutions (CT and BC), and only one chimeric architecture was robustly active in both *E.coli* and HeLa (BC; Fig. 7A,B).

Active fusions between the phiC31 and TG1 integrases proved to be surprisingly difficult to make. Despite the high similarity of their catalytic domains (∼70%; supplementary material Table S5) and *att*P sites (∼80%; supplementary material Fig. S3), the only active full-domain-swap hybrids that we could produce were of the CT variety. TC chimeras with a full TG1 integrase catalytic domain were not active on any of the tested parental or hybrid *att*-site pairings. This was an unexpected result because, as evidenced by certain BC hybrids, the C-terminal domain of phiC31 integrase is able to function with the catalytic domain of phiBT1 integrase, a much more distant relative (∼40% similar; supplementary material Table S5). One possible explanation for these results is that a structurally-incompatible mutation has arisen in the TG1 integrase catalytic domain, because the phiC31 counterpart can still function with the phiC31 and TG1 C-terminal domains. Another possibility is that a change in the phiC31 C-terminal domain is responsible, but we feel that this is less likely, as the distantly-related phiBT1 catalytic domain remains compatible with it (e.g. BC hybrids can function).

While we were able to demonstrate clear CT{8,8} activity in *E. coli*, our CT{8,8} hybrid expression plasmid performed poorly in HeLa, HEK-293 and NIH-3T3 cells. Whereas a similar TG1 integrase vector (supplementary material Table S4) induced valid GFP signal above background for all tested TcT att-site pairings in HeLa (P0 × B0, P3 × B0, P0 × B3 and P3 × B3), we were only able to confirm the expected CT{8,8} TcT P3 × B3 activity in HeLa via PCR amplification and sequencing of the attL and attR junctions (supplementary material Fig. S4). GFP fluorescence was never detected above background for the CT{8,8} reaction, so it is likely to have only been mediated at trace levels in HeLa.

The reason(s) for this severe inhibition of CT{8,8} activity in mammalian cells are not clear. We investigated three potential problems that can be encountered when expressing prokaryotic proteins in mammals – aberrant mRNA splicing/stability, protein mis-localization and protein insolubility – all to no avail. Neither codon optimization nor addition of an SV40 NLS managed to rescue activity, and solubility of an HA-tagged CT{8,8} was confirmed via a western blot (data not shown). Several potential sources of CT{8,8} inhibition remain, but are outside the scope of our serine integrase chimera study. One possibility is that the majority of synthesized CT{8,8} protein is mis-folded in mammals due to lack of ClpB/Hsp104 chaperone activity. While present in *E. coli* and the vast majority of eukaryotes, metazoans completely lack a ClpB/Hsp104 homolog or functional equivalent (DeSantis and Shorter, 2012; Murray and Kelly, 2012), so it is possible that nearly all of CT{8,8} exists in an inhibitory conformation (that remains soluble). A second potential explanation for CT{8,8} activity suppression in HeLa is synergistic inhibition. For example, a protein bound to the phiC31 integrase catalytic domain might form an inhibitory complex with a factor attached to the TG1 C-terminal domain, leading to blockage of CT hybrid function, but not phiC31 or TG1 integrase activity. A third potentially contributing source of inhibition for CT{8,8} recombination in HeLa is the heightened difficulty of the pMF inversion assay. Because the E. coli pFlip assay is qualitative, we could not quantifiably compare it to pMF recombination. However, it is clear that our CMV-driven pMF platform is more difficult to invert than our previously described tet-driven system (pCSkI + PB-OFF: ∼80% efficiency in HeLa) (Farruggio et al., 2012), so it is possible that a stronger-promoter platform would have produced a clear CT{8,8} TcT P3 × B3 recombination signal.

Chimeras produced from the phiBT1 and phiC31 integrases also proved to be surprising. While we were unable to produce any functional CB hybrids, a few of our BC chimeras exhibited activity in *E. coli*, and one BC hybrid also performed well in HeLa (Fig. 7, Fig. 5B). The activity of BC chimeras was an unexpected result given our experience with TG1 integrase, as the phiBT1 and phiC31 catalytic domain sequences are several-fold more dissimilar when judged by alignment score (supplementary material Table S5).

In *E. coli*, BC{−1} exhibited a broader activity profile than the CT hybrids, since it was able to react both wild-type and hybrid *att*-sites (Fig. 4C,D). This profile carried over to HeLa, where general BC{−1} expression plasmid performance (max ∼37% eff.) was competitive with both parental enzyme vectors (∼40–46% eff.; Fig. 5B). Our BC{−1} integrase plasmid induced recombination of hybrid *att*-sites with less efficiency than the wild-type phiC31 P0 × B0 reaction (∼20–30% vs ∼37%), but its hybrid-site performance was still superior to that of the phiC31 vector for the three tested combinations (∼1–24% eff. Fig. 5B).

Like its parents, BC{−1} was also able to mediate recombination into endogenous pseudo-sites in HeLa (Fig. 6D). The pseudo-*att*P recombination performance of our BC{−1} expression plasmid was competitive with the phiC31 integrase vector, however, unlike phiC31, BC{−1} proved to also be capable of integration into pseudo-*att*B sites (Fig. 6D). This feature may have been inherited from phiBT1 integrase, which is also capable of pseudo-*att*B recombination (our unpublished observations). Recognition of both pseudo site types may preclude usage of BC{−1} for genomic engineering due to safety concerns, as recombination between said sites could lead to unwanted inversions, deletions and/or translocations. However, we have evidence that the pseudo-site reactivity of phiC31 integrase can be lowered to background levels in mammalian cells without also inhibiting wild-type recombination efficiency (unpublished observations; to be addressed in a future study), so it might be possible to create an improved variant of BC{−1} integrase for use in genomic engineering applications.

With this study, we have shed light on the degree of structural compatibility that exists between the catalytic and C-terminal domains of the phiC31, phiBT1 and TG1 integrases. Furthermore, we have laid the groundwork for the use of hybridization to create serine integrases with novel specificities. We describe chimeras with rationally altered and/or broadened reactivity relative to their parents, all produced via catalytic domain swaps.

While this manuscript was in preparation, a new serine integrase structure, PDB ID 4KIS, was published by Rutherford et al. The structure is of an N-terminal truncation mutant of LI integrase (LI-int) bound to an A118 integrase (A118-int) attP half-site (Rutherford et al., 2013).

This structure reveals the presence of two novel DNA binding domains in the C-terminal domain of LI-int: a ∼100 residue mixed α/β “recombinase” domain (RD) that is adjacent to αE and a ∼180 residue zinc-beta ribbon domain (ZBRD) downstream of the RD. While the region corresponding to the LI-int RD in C31-int has been previously identified as a DNA binding domain (McEwan et al., 2011), Rutherford et al. have provided the first direct evidence that the downstream ZBRD is in fact a second DNA-binding domain (and is not, e.g., just important for optimal serine integrase activity). The importance of zinc-coordination by the LI-int ZBRD remains to be determined, since it has been reported recently that A118-int activity (LI and A118-int are 98% identical) does not appear to be inhibited in the presence of a zinc-chelator (Mandali et al., 2013).

In addition to identifying the LI-int DNA binding domains, Rutherford et al. have demonstrated that the two linker segments that flank the recombinase domain are likely to be involved in att-site interaction. If these linkers also play this role in the phiC31 family of integrases that we used to make hybrids, then this revelation by Rutherford et al. offers a potential explanation for why many of the hybrids that were fused in the αE-RD linker region did not function (supplementary material Fig. S5; e.g. CB{2}, CT{0}, TC{−1}, etc.).

While avoidance of these linkers may increase the likelihood of obtaining an active hybrid, it is clear that other barriers remain, as several hybrids that were fused in other regions were also inactive (supplementary material Fig. S5; e.g. CB{−82}, TC{−43}, TC{−7}, etc.).

In combination with the work that we have described here, this new structural information strengthens the viability of using hybridization to develop serine integrases with novel specificities. While it is likely that directed evolution will still be necessary to optimize the stringency of chimeras for endogenous *att*-site targets, the mixing and matching of domains obtained from the ever-growing list of putative large serine recombinases may make this approach tractable for the first time.

## MATERIALS AND METHODS

### Plasmid construction

Integrase activity tests in *E. coli* were performed with all-in-one plasmids given the prefix “pFlip” (supplementary material Fig. S1A). These vectors were cloned via a series of PCR, exonuclease, restriction digest and ligation steps. The PCR-amplified functional elements that are common to all pFlip plasmids were sourced from pET-50b (Novagen). These elements include the repressor of primer (rop) cassette, ColE1 replication origin, kanamycin-resistance (kanR) cassette, and lac repressor (lacI) ORF. All *att*-sites and remaining bacterial regulatory sequences were synthesized *in vitro* (oligos from Invitrogen). Phusion polymerase, lambda exonuclease, T4 DNA ligase and all restriction enzymes used for pFlip cloning were obtained from NEB. Optikinase was purchased from USB. Sequences of the 50 nt *att*-sites and hybrid integrase cloning primers have been provided in the supplementary material (supplementary material Tables S2 and S6, respectively).

In HeLa cells, we used separate vectors for inversion detection and protein expression. Our inversion plasmids – given the prefix “Pmf” (supplementary material Fig. S1B) – were constructed in a manner similar to the pFlip vectors, i.e. a series of PCR, exonuclease, restriction digest and ligation steps. All PCR-amplified functional elements used in the pMF plasmids were derived from pEGFP-C1 (Clontech). These elements include the CMV promoter, enhanced green fluorescent protein ORF (EGFP) and kanamycin/neomycin resistance cassette. All cloned *att*-sites are 50 nt in length (supplementary material Table S2) and were synthesized *in vitro* (oligos form Invitrogen).

Integrase expression vectors – given the prefix “pN1t8” (supplementary material Fig. S1C,D) – were constructed via standard PCR amplification, restriction digest and ligation steps. The functional elements common to all pN1t8 vectors include a tet promoter, SV40 early 3′ UTR and kanamycin-resistance cassette, which were PCR-amplified from the PB-TET-MKOS (Woltjen et al., 2009), pCMVInt (Groth et al., 2000) and pET-50b (Novagen) vectors, respectively. For all HeLa experiments presented in this article, integrases that carry a C-terminus SV40 nuclear localization signal (NLS) were used (Kalderon et al., 1984).

Pseudosite integration donor vectors (Fig. 6B,C) were cloned via standard PCR and restriction digest protocols (all enzymes from NEB). All constructed donors consist of the pDB2 (Keravala et al., 2006b) plasmid backbone with the appropriate 50-bp hybrid *att*-site (supplementary material Table S2) cloned in place of phiC31 *att*B.

### E. coli plasmid inversion assay

To perform activity tests in *E. coli*, we assembled pFlip vectors with the desired integrase and *att*-site combinations via *in vitro* ligation, and then transformed these ligations into NEB 10β cells (C3019H) using their high-efficiency protocol. The transformed cells were plated on X-gal (70 µg/ml) IPTG (80 µM) kanamycin (50 µg/ml) LB agar plates after 1 hour of outgrowth at 37°C in SOC. Plates were incubated at 37°C for 16 hours and were then visually assessed for the presence of blue colonies. For each integrase-*att*-site pairing, minipreps from at least two separate colony outgrowths were sequenced (Sequetech, Mountain View) to confirm the validity of all PCR-amplified integrase and/or *att*-site segments. In addition, all recombined junctions were confirmed to be as expected via sequencing of plasmids from at least two colonies (Sequetech).

### HeLa plasmid inversion assay

To perform the transient recombination assay in HeLa (ATCC CCL-2), the cells were grown to sub-confluence (60–80%) in DMEM (Cellgro 10-013-CV) supplemented with 9% FBS (Gemini Benchmark 100-106), 1× GlutaMAX (Invitrogen 35050-061), 1% penicillin/streptomycin (Invitrogen 15140-122), and 1.5 µg/ml doxycycline (Sigma D9891) in 24-well plates. Transfection was performed in triplicate, overnight, with 3 µg of DNA per well using Xfect (Clontech) at a 0.3 µl:1 µg polymer to DNA ratio. For all transfections, 500 ng of the PB-CA-rtTA tet-promoter activation plasmid (Woltjen et al., 2009; Woltjen et al., 2011), and 580 ng of the appropriate pMF inversion-reporter vector were included in the DNA mix. For transfections that included an integrase expression plasmid, approximately 490 femtomoles of the respective pN1t8 vector was included. The pCS plasmid (Olivares et al., 2002) was used as a filler to bring all DNA mixtures to 3 µg/well. Fluorescent cells were counted and analyzed 48 hours post transfection with a flow cytometer at the Stanford Shared FACS Facility (custom ‘Scanford’ FACScan analyzer; 10,000 events per sample). GFP expression from the pEGFP-C1 plasmid was used as a proxy for transfection efficiency, which ranged from 45–80%.

To validate the recombined *att*L and *att*R junctions, they were PCR amplified (primers in supplementary material Table S7) and sequenced using our previously described method (Farruggio et al., 2012). Briefly, we recovered each transfected plasmid mixture after 48 hours using a miniprep protocol for mammalian cells (Siebenkotten et al., 1995) and then used these purified vectors as PCR templates (HotStarTaq Plus, Qiagen). The junction amplicons were column-purified (MinElute, Qiagen) and then directly sequenced (Sequetech).

### HeLa pseudosite integration assay

To test for integration into pseudosites, HeLa cells were grown in 24-well plates to sub-confluency (60–80%) using the same medium described for the plasmid inversion assay, and were then transfected in triplicate with a ternary mixture consisting of polyethylenimine (PEI), γ-polyglutamic acid (PGA) and plasmid (pDNA) (Kichler et al., 2005). To form the PEI-PGA-pDNA complexes, 375 ng pDNA, 170 ng PGA and 2.18 µg PEI were combined in order, brought to a final 50 µl volume with 150 mM NaCl, mixed vigorously via pipetting and then left to incubate at room temperature for 15 minutes. Each 375 ng pDNA mixture consisted of 6 ng *att*-site donor plasmid (e.g. Fig. 6A–C), 63 ng PB-CA-rtTA (tet-promoter activation plasmid) and 306 ng of the desired integrase expression plasmid or negative control pCS vector. Transfection efficiency, which we estimated via GFP expression from the pDB2 plasmid (Keravala et al., 2006b), ranged from 30–40%. After 24 hours, the cells were trypsinized and transferred to 12-well plates (only 50% of each well transferred). G418 selection (0.8 mg/ml) was started the next day (48 hr after transfection), and was maintained for ∼2 weeks. Colonies were stained with Neutral Red and counted manually. PEI was obtained from Polysciences, Inc. (23966-2), PGA from Sigma (G0421), G418 from Invitrogen (10131) and Neutral Red from Sigma (N4638).

## Supplementary Material

Supplementary Material
