# Supplementary material for: Serine integrase chimeras with activity in E. coli and HeLa cells
Source: Biol Open. 2014 Sep 12;3(10):895–903. doi: 10.1242/bio.20148748 (PMC4197438; doi:10.1242/bio.20148748)
Supplement: Supplementary Material [file supp_3_10_895__index.html]

Serine integrase chimeras with activity in E. coli and HeLa cells — Supplementary Material 

# Serine integrase chimeras with activity in *E. coli* and HeLa cells

## bio.20148748 Supplementary Material

**Files in this Data Supplement:**

- Supplementary Material - Alfonso P. Farruggio and Michele P. Calos doi: 10.1242/bio.20148748
