# Supplementary material for: Serine integrase chimeras with activity in E. coli and HeLa cells
Source: Biol Open. 2014 Sep 12;3(10):895–903. doi: 10.1242/bio.20148748 (PMC4197438; doi:10.1242/bio.20148748)
Supplement: Supplementary Material [file supp_bio.20148748_bio.20148748-s1.pdf]

Biology Open (2014) 000, 1–9 doi:10.1242/bio.20148748

Supplementary Material

Alfonso P. Farruggio and Michele P. Calos doi: 10.1242/bio.20148748

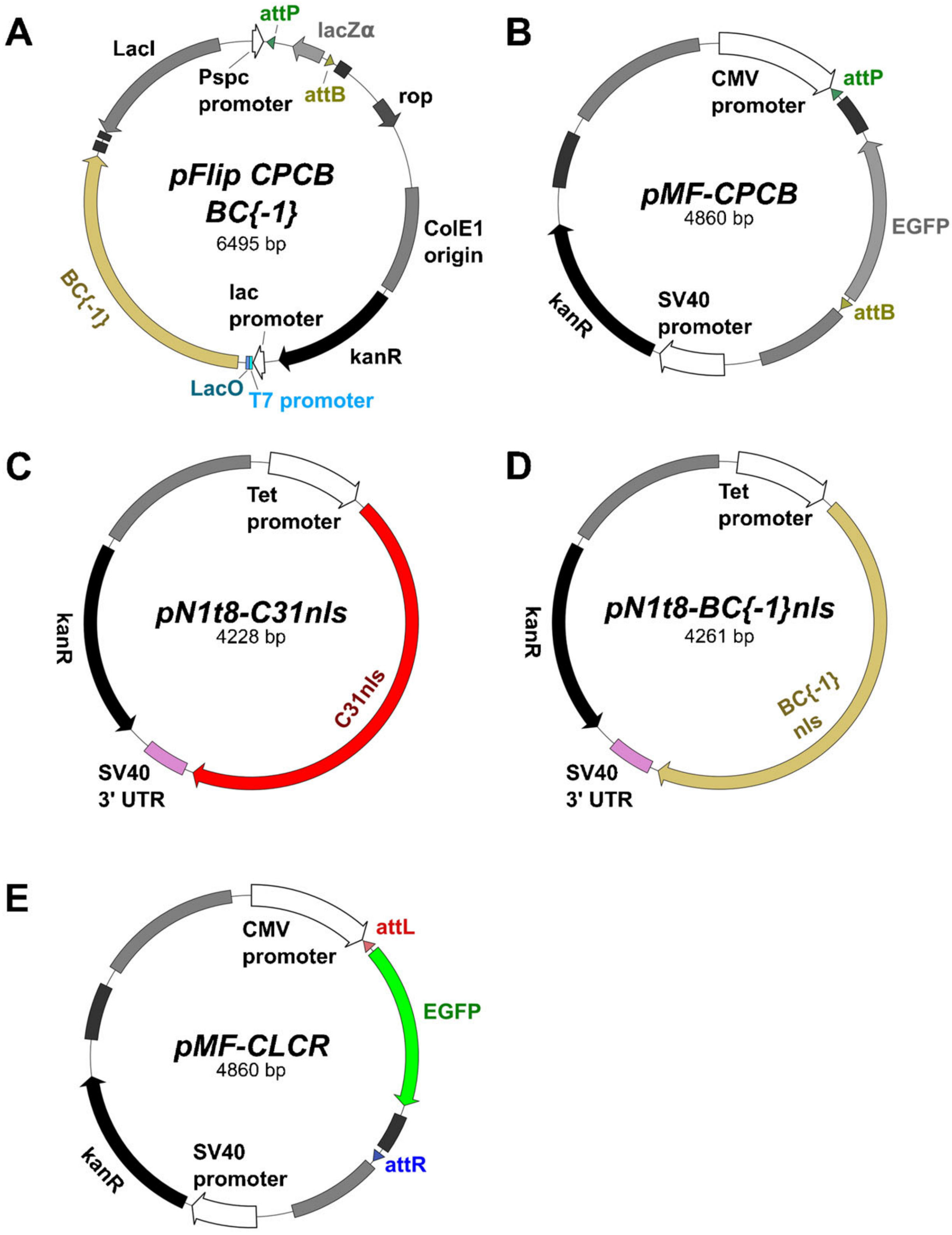

Fig. S1. Detailed maps of representative inversion and protein expression plasmids. (A) pFlip CPCB BC{−1}, used to test BC{−1} integrase PxB reactivity on wildtype phiC31 att-sites in E. coli. (B) pMF-CPCB, the mammalian PxB inversion plasmid with wildtype phiC31 attP and attB sites. (C) pN1t8-C31nls and (D) pN1t8-BC{−1}nls, the mammalian vectors used for tet-driven expression of phiC31 and BC{−1} integrases, respectively. (E) pMF-CLCR, recombination product of pMF-CPCB.

Biology Open

Biology Open (2014) 000, 1–9 doi:10.1242/bio.20148748

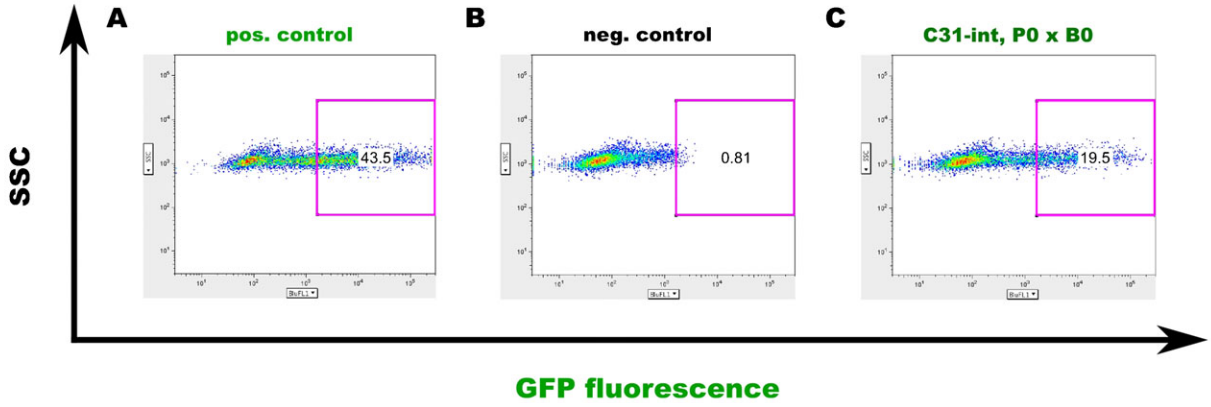

Fig. S2. Representative flow cytometry plots from HeLa inversion experiment. The vertical and horizontal axes are side-scatter (SSC) and green fluorescence, respectively. Flow cytometry was performed 48 hours after transfection. Shown here are the results from HeLa cells exposed to three different plasmid cominbations: (A) pCS + pMF-CLCR (43.5% in GFP+ gate), (B) pCS + pMF-CPCB (0.81% GFP+) and (C) pN1t8-C31nls + pMF-CPCB (19.5% GFP+).

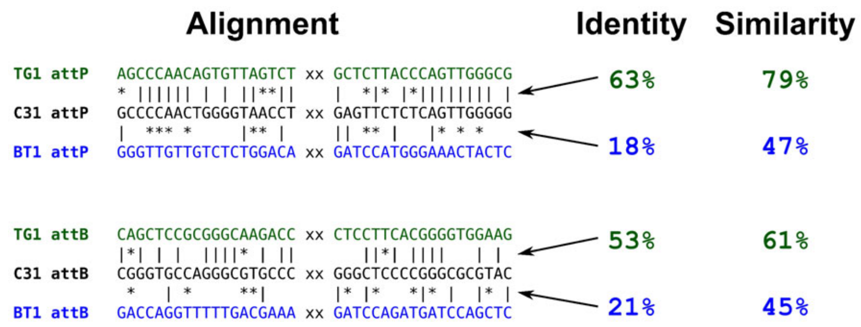

Fig. S3. Wildtype att-site alignments. The 40 base core att-sites of TG1 and BT1 integrase were aligned without gaps to their phiC31 counterparts. The percentage of identical and similar bases were calculated without consideration of the dinucleotide crossover core (indicated by 'xx') – only 38 nucleotides were compared in each alignment. We consider two bases to be similar if they belong to the same class of nucleobase (purine or pyrimidine). An asterisk is used in the alignment to indicate bases that are similar but not identical.

A

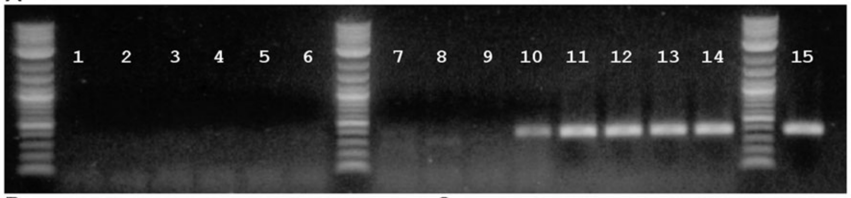

B

	P0 x B0	P0 x B3	P3 x B0	P3 x B3
pCS	3	4	5	6
CT{8,8}	7	8	9	10
TG1	11	12	13	14

C

	P0 x B0	P0 x B3	P3 x B0	P3 x B3
pCS	x	x	x	x
CT{8,8}	x	x	x	+
TG1	+	+	+	+

Fig. S4. PCR amplification of TcT attR junctions. An EGFP inversion assay was performed in HeLa cells on four pMF-TcT vectors: pMF-TcT-P0B0, pMF-TcT-P0B3, pMF-TcT-P3B0 and pMF-TcT-P3B3. The pCS negative control plasmid or one of two integrase expression vectors (pN1t8-CT{8,8}nls; pN1t8-TG1nls) was co-transfected with each pMF-TcT plasmid. PCR for the recombination junctions was performed two days after transfection. (A) PCR results for the TcT attR junctions. attL PCRs were also performed and gave the same results. Lanes 1 and 2: water and naive HeLa cells, respectively (negative controls). Ladder: NEB 2-log (N3200S). (B) Explanation of lanes 3–14. Lane 15: pCS + pMF-TcT-L3R3 (positive control). (C) Summary of PCR results. The symbols 'x' and '+' are used to indicate samples that gave negative and positive PCR results, respectively. All PCR amplicons were column-purified and directly sequenced to confirm their identities.

Biology Open (2014) 000, 1–9 doi:10.1242/bio.20148748

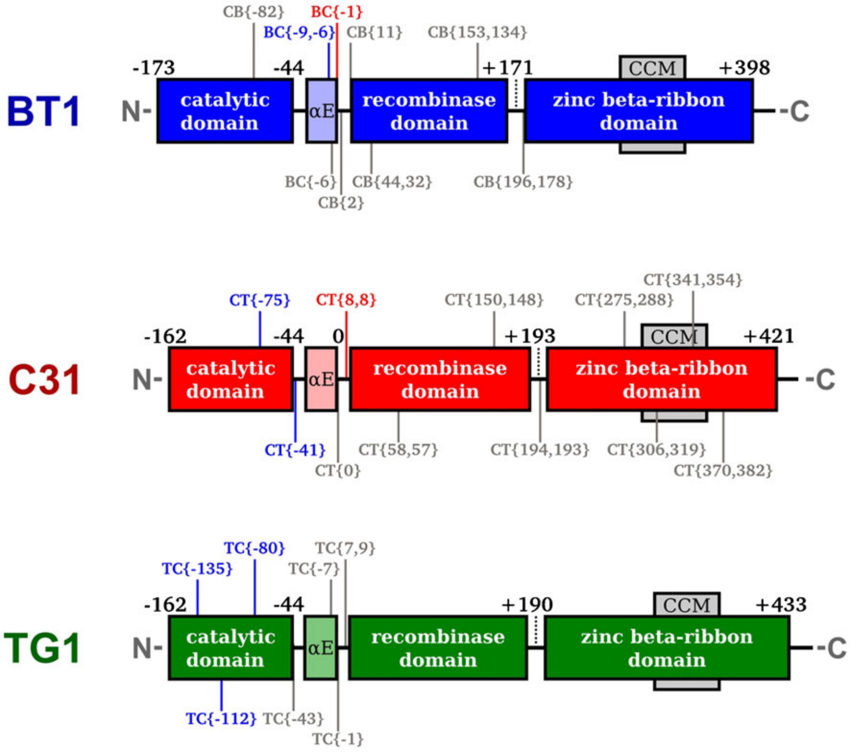

Fig. S5. Map of chimera fusions. Predicted boundaries of the catalytic, recombinase and zinc-beta ribbon domains of the phiBT1, phiC31 and TG1 integrases are shown here. Secondary structure prediction (Jpred3) and the LI-phiC31 integrase alignment provided in Rutherford et al. were used to predict domain bounds (Rutherford et al., 2013). In addition, alpha helix E (αE) and the coiled-coil binding motif (CCM) are depicted. The bounds for αE were determined via secondary structure prediction (Jpred3) and the CCM ranges were obtained from an alignment published previously (Rowley et al., 2008). The fusion points of all hybrids discussed in this work have been indicated in either the N- or C-terminal parental integrase used to make the chimera. Hybrids with a gray font color were not active in any of the tests that they were subjected to (see text). Blue font coloring is used to indicate chimeras that were active in E. coli and red coloring has been used for hybrids that were shown to be active in HeLa (not all hybrids were tested in HeLa).

Table S1. Serine integrase domain size estimates. The secondary structures of 18 characterized serine integrases were predicted with Jpred 3 on Feb 13, 2014 (Cole et al., 2008). The ''catalytic'' and ''CTD'' columns are the number of amino acids upstream and downstream of the predicted alpha-helix E (''αE''), respectively. The ''simple Jnet'' output of Jpred was used for αE length estimation.

Name	Accession	Total length	Catalytic	αE	CTD
A118	Q9T193	452	115	31	306
U153	Q8LTD8	452	113	33	306
MR11	A8R7E9	458	113	33	312
ΦFC1	Q9XJF6	464	118	32	314
BL3	D7RWL2	465	119	33	313
ΦRV1	O06604	469	121	31	317
R4	Q37839	469	117	32	320
Φ370.1	Q9A0R5	471	118	32	321
Wβ	Q2LIF8	481	118	33	330
TP901-1	Q38184	485	120	31	334
Bxb1	Q9B086	500	119	32	349
Peaches	C9DC17	507	132	35	340
Bxz2	Q857I8	522	129	35	358
SPBc	O64015	545	130	34	381
ΦK38	Q0KKA9	552	147	33	372
ΦBT1	Q858X3	594	142	32	420
ΦC31	Q9T221	605	131	32	442
TG1	Q0KKA7	619	130	33	456
**mean**		506.1	124.0	32.6	349.5
**median**		483.0	119.5	32.5	332.0
**min**		452	113	31	306
**max**		619	147	35	456

Biology Open

Biology Open (2014) 000, 1–9 doi:10.1242/bio.20148748

Table S2. Hybrid and wildtype *att*-sites. Sequences of the 50 base *att*-sites used in our constructs. The phiC31 and phiBT1 *att*B sequences were derived from the wildtype *Streptomyces coelicolor* ('*S. coel.*') bacterial attachment sites (Gregory et al., 2003; Rausch and Lehmann, 1991), and the TG1 *att*B sequence is from *Streptomyces Avermitilis* ('*S. aver.*') (Morita et al., 2009). To simplify our experiments, phiBT1 *att*P and *att*B sites with a 'TT' crossover core – instead of the wildtype 'GT' core – were used. The dinucleotide crossover sequence does not affect serine integrase recombination efficiency, and this specific change has been demonstrated to not influence the activity of phiBT1 integrase (Zhang et al., 2010).

Name	***Sequence***
ΦBT1 *att*P (BcB-P0)	5-GTGCTGGGTTGTTGTCTCTGGACA tt GATCCATGGGAAACTACTCAGCAC-3
ΦC31 *att*P (CbC-P0, CtC-P0)	5-gtagtgccccaactggggtaacct tt gagttctctcagttgggggcgtag-3
CbC-P3	5-gtagtgccccaactggggtaaAcA tt gaTttctctcagttgggggcgtag-3
CbC-P6	5-gtagtgccccaactggggtGGAcA tt gaTCCAtctcagttgggggcgtag-3
BcB-P3	5-GTGCTGGGTTGTTGTCTCTGGcCt tt GAgCCATGGGAAACTACTCAGCAC-3
BcB-P6	5-GTGCTGGGTTGTTGTCTCTaacCt tt GAgttcTGGGAAACTACTCAGCAC-3
TG1 *att*P (TcT-P0)	5-GTTCCAGCCCAACAGTGTTAGTCT tt GCTCTTACCCAGTTGGGCGGGATA-3
TcT-P3	5-GTTCCAGCCCAACAGTGTTAGcCT tt GagCTTACCCAGTTGGGCGGGATA-3
TcT-P6	5-GTTCCAGCCCAACAGTGTTAacCT tt GagtTcACCCAGTTGGGCGGGATA-3
CtC-P3	5-gtagtgccccaactggggtaaTct tt gCTttctctcagttgggggcgtag-3
CtC-P6	5-gtagtgccccaactggggtaGTct tt gCTCtTtctcagttgggggcgtag-3
ΦBT1 *att*B (BcB-B0; *S. coel.*)	5-TCCTTGACCAGGTTTTTGACGAAA tt GATCCAGATGATCCAGCTCCACAC-3
ΦC31 *att*B (CbC-B0, CtC-B0; *S. coel.*)	5-cggtgcgggtgccagggcgtgccc tt gggctccccgggcgcgtactccac-3
CbC-B3	5-cggtgcgggtgccagggcgtgAAA tt gATctccccgggcgcgtactccac-3
CbC-B6	5-cggtgcgggtgccagggcACgAAA tt gATcCAcccgggcgcgtactccac-3
BcB-B3	5-TCCTTGACCAGGTTTTTGACGccc tt GggCCAGATGATCCAGCTCCACAC-3
BcB-B6	5-TCCTTGACCAGGTTTTTGgtGccc tt GggCtcGATGATCCAGCTCCACAC-3
TG1 *att*B (TcT-B0; *S. aver.*)	5-TCGATCAGCTCCGCGGGCAAGACC tt CTCCTTCACGGGGTGGAAGGTCGG-3
TcT-B3	5-TCGATCAGCTCCGCGGGCAAGcCC tt gggCTTCACGGGGTGGAAGGTCGG-3
TcT-B6	5-TCGATCAGCTCCGCGGGCgtGcCC tt gggCTcCACGGGGTGGAAGGTCGG-3
CtC-B3	5-cggtgcgggtgccagggcgtgAcc tt CTCctccccgggcgcgtactccac-3
CtC-B6	5-cggtgcgggtgccagggcAAgAcc tt CTCctTcccgggcgcgtactccac-3

Table S3. Concise summary of hybrid integrase activity test results. Chimeras that were able to perform the PxB reaction for at least one of the nine *att*-site combinations in *E. coli* (Fig. 3) are considered active. In HeLa, only the CT{8,8} and BC{−1} chimeras were subjected to a complete battery of tests, which consisted of four *att*-site recombination pairs (P0 × B0, P3 × B0, P0 × B3 and P3 × B3; TcT and CbC sites, respectively).

	Hybrids		
Active in E. coli	CT{−75}	CT{−41}	CT{8,8}
	TC{−135}	TC{−112}	TC{−80}
	BC{−9,−6}	BC{−1}	
No evidence of activity in E. coli	CT{0}	CT{58,57}	CT{150,148}
	CT{194,193}	CT{275,288}	CT{306,319}
	CT{341,354}	CT{370,382}	
	TC{−43}	TC{−7}	TC{−1}
		TC{7,9}	
		BC{−6}	
	CB{−82}	CB{2}	CB{11}
	CB{44,32}	CB{153,134}	CB{196,178}
Active in HeLa	CT{8,8}[a]		BC{−1}

[a]In HeLa, CT{8,8} hybrid activity could only be detected via PCR amplification of the *att*L and *att*R junctions (sequence verified).

Biology Open (2014) 000, 1–9 doi:10.1242/bio.20148748

Table S4. Summary of pMF and pN1t8 plasmid alignments. (A) Results of pMF vector alignments. Four EGFP flipper plasmids were aligned to each other using Nucmer version 3.1 (MUMmer package version 3.23; 'breaklen'=5): pMF-CPCB (CbC-00), pMF-CbC-P3B3 (CbC-33), pMF-CLCR (LR) and pMF-BT1 (BT1) (Kurtz et al., 2004). Percent identities are shown here, and were calculated by dividing the total number of identical bases in each alignment by the vector size (all aligned plasmids have same 4860 bp length). (B,C) Results of pN1t8 plasmid alignments. Vectors used to express the indicated integrase proteins for the HeLa inversion experiment were aligned using the EMBOSS Needle program with the following parameters: gap_open=10, gap_ext=0.5, end_gap_penalty=true, end_gap_open=10, end_gap_extend=0.5. Percent identities are shown here.

A **EGFP inversion plasmids**

	CbC-00	CbC-33	LR*	BT1
CbC-00	100%	99.7%	100%	98.0%
CbC-33		100%	99.7%	98.0%
LR			100%	98.0%
BT1				100%

B **Integrase expression plasmids**

	BC{−1}	C31	BT1
BC{−1}	100%	92.5%	81.0%
C31		100%	74.5%
BT1			100%

C

	CT{8,8}	C31	TG1
CT{8,8}	100%	85.0%	94.9%
C31		100%	80.3%
TG1			100%

*The Nucmer alignment algorithm correctly detected and aligned the inverted EGFP segment for all LR alignments.

Table S5. Summary of phiC31 catalytic domain alignments. The catalytic regions (residues upstream of Jpred 3 predicted αE) of 17 characterized integrases were individually aligned to the phiC31 integrase catalytic domain with the EMBOSS Needle algorithm (using the BLOSUM62 matrix, with gap-open, gap-extend, end-gap-open and end-gap-extend penalties of 10, 0.5, 10 and 0.5, respectively). The results have been sorted by alignment score.

Integrase	Score	Identity [%]	Similarity [%]	Gaps [%]
ΦC31	658.0	100	100	0
TG1	381.0	61.1	72.5	0.8
ΦBT1	107.5	24.8	40.0	11.7
TP901	62.5	19.7	35.9	23.2
BL3	57.5	22.6	37.6	12.0
Φ370.1	56.0	23.4	41.6	18.2
SPBc	55.0	23.8	38.1	22.4
ΦFC1	50.0	24.6	39.6	14.2
Wβ	50.0	23.4	41.8	23.4
ΦRv1	33.5	18.9	33.8	29.7
A118	32.5	23.1	35.8	16.4
U153	28.5	23.0	35.3	24.5
MR11	24.0	19.5	38.3	16.5
R4	12.5	18.4	31.9	24.1
Bxz2	12.0	16.9	31.7	16.9
ΦK38	5.5	23.6	35.0	22.9
Bxb1	2.5	21.0	32.6	18.8
Peaches	−2.5	15.8	29.7	40.6

Biology Open (2014) 000, 1–9 doi:10.1242/bio.20148748

Table S6. Hybrid integrase cloning primers. Oligos used for construction and cloning of BC{−1}, CT{−75}, CT{8,8} and TC{−80} hybrid integrases into pFlip backbone. Phusion polymerase (NEB) was used for all PCRs with the listed primers. The DNA templates used for PCR of the segments were as follows: phiBT1 integrase for BC-A; phiC31 integrase for BC-B, CT88-A, CT-A and TC-B; TG1 integrase for CT88-B, CT-B and TC-A. The BsaI type IIS restriction enzyme (NEB) was used to create seamless cohesive overhangs between the amplified phiBT1, phiC31 and TG1 integrase segments. All cloning oligos were synthesized by Invitrogen.

Hybrid name	Segment name	Primer name	Primer sequence
BC{−1}	BC-A	Nde_BT1_fwd	5-cttcttCATATGtcgcccttcatcgctcccgac-3
		BsaI_BCC_BT1_rev	5-gttgttGGTCTCtGCCACCCAGCGCGGCAATCTCGTCTTTCGC-3
	BC-B	BsaI_BCC_C31_fwd	5-gttgttGGTCTCtTGGCtacgtcggcgggaaggcgccttacggcttc-3
		AvrII(BsaI)_C31_rev	5-gaagaaGGTCTCTCTAGGATTATTATTAcgccgctacgtcttccgtgc-3
CT{−75}	CT88-A	NdeI_72_C31_fwd	5-cttcttCATATGgacacgtacgcgggtgcttacgaccg-3
		88_CTC_BsaI_C31_rev	5-GTTGTTGGTCTCTtgatcatgttgagccgcccg-3
	CT88-B	88_CTC_BsaI_TG1_fwd	5-CAACAAGGTCTCTatcattgttcattacatttc-3
		AvrII(BsaI)_72_TG1_rev	5-gaagaaGGTCTCTCTAGGATTATTATTAcgccgccgctgtgaac ccgttcag-3
CT{8,8}	CT-A	NdeI_72_C31_fwd	5-cttcttCATATGgacacgtacgcgggtgcttacgaccg-3
		S+0_BsaI_C31_rev	5-CAACAAGGTCTCTgccgtaaggcgccttcccgccgacgtacc-3
	CT-B	S+0_BsaI_TG1_fwd	5-CAACAAGGTCTCTcggcttcgacacggtcgaggaaatggt tccg-3
		AvrII(BsaI)_72_TG1_rev	5-gaagaaGGTCTCTCTAGGATTATTATTAcgccgccgctgtgaac ccgttcag-3
TC{−80}	TC-A	NdeI_TG1_fwd	5-GAAGAACCATATGgtcattctggcaggcggc-3
		88_TC__BsaI_TG1_rev	5-GTTGTTGGTCTCTtgatcatgttcatttcccg-3
	TC-B	88_TC__BsaI_C31_fwd	5-CAACAAGGTCTCTatcattgtctatgacgtgtc-3
		AvrII(BsaI)_C31_rev	5-gaagaaGGTCTCTCTAGGATTATTATTAcgccgctacgtcttccgtgc-3

Table S7. Primers used for amplification of *att*L and *att*R junctions in HeLa plasmid-inversion assay

Amplicon	*Name*	*Sequence*
*att*L	NheI_pMF_attL_fwd	5-GCAGAGCTGGTTTAGTGAACCGTCAGATCC-3
*att*L	BamHI_pMF_attL_rev	5-GGTATGGCTGATTATGATCAGTTATCTAGATCCGGT-3
*att*R	BamHI_pMF_attR_fwd	5-TCGACGGTACCGCGGGCCCG-3
*att*R	EcoRI_pMF_attR_rev	5-CGCGAATTTTAACAAAATATTAACGCTTACAATTTACGC-3
